# Supplementary material for: Functions and trafficking mechanisms of RIC-8 in C. elegans and mammalian cilia
Source: Mol Biol Cell. 2026 Jul 27;37(8):br26. doi: 10.1091/mbc.E25-12-0598 (PMC13428341; doi:10.1091/mbc.E25-12-0598)
Supplement: Supplementary file 1 [file mbc-37-br26-s001.pdf]

Supplemental Materials

Molecular Biology of the Cell

Campagna *et al*.

Supplementary Figure 1. The RVxP motif precludes RIC-8 from localizing to the distal cilium. (A) Alignment of the *C. elegans* RIC-8 (NP 001023561) and human RIC8A (NP 068751) and RIC8B (NP 001317074) sequences showing presence of the RVIP motif (orange box) in the *C. elegans* protein. (B and C) Images (B) and quantification (C) of RIC-8^{WT}::TagRFP and RIC-8$^{\Delta RVIP}$::TagRFP localization in phasmid neurons of WT adults. Arrowheads: TZ; arrows: distal boundary of RIC-8::TagRFP signal. d: dendrite. Scale: 5 μm. * Different from wild type at $p<0.05$ (Mann-Whitney test).

Supplementary Figure 2. Ciliation is reduced in *RIC8A* and *RIC8B* KD RPE-1 cells. (A) Immunofluorescence images of fixed RPE-1 cells transfected with the indicated siRNAs and stained with anti-acetylated α-tubulin antibody and DAPI. siCTRL: non-targeting siRNA. Scale: 20 μm. (B – C) Quantification of ciliation (B) and relative RIC8A and RIC8B mRNA levels (C) in RPE-1 cells transfected with the indicated siRNAs. (D) Quantification of ciliation in untransduced RPE-1 cells and RPE-1 cells transduced with *RIC8B*-overexpressing lentivirus after transfection with siControl or si*RIC8B* siRNA #1. Each data point in (B) and (D) represents one KD experiment; biological replicates are shown in different shades of the corresponding color in (D). *** Different from siCTRL (B) or between bracketed conditions (D) at $p<0.001$ (Fisher's exact test). (E) Quantification of relative mRNA levels in RPE-1 cells transfected with the indicated siRNAs. ** Different between bracketed conditions at $p<0.01$ (Welch's t test). Summary data in (C) and (E) represent three biological replicates per condition, with three technical replicates each.

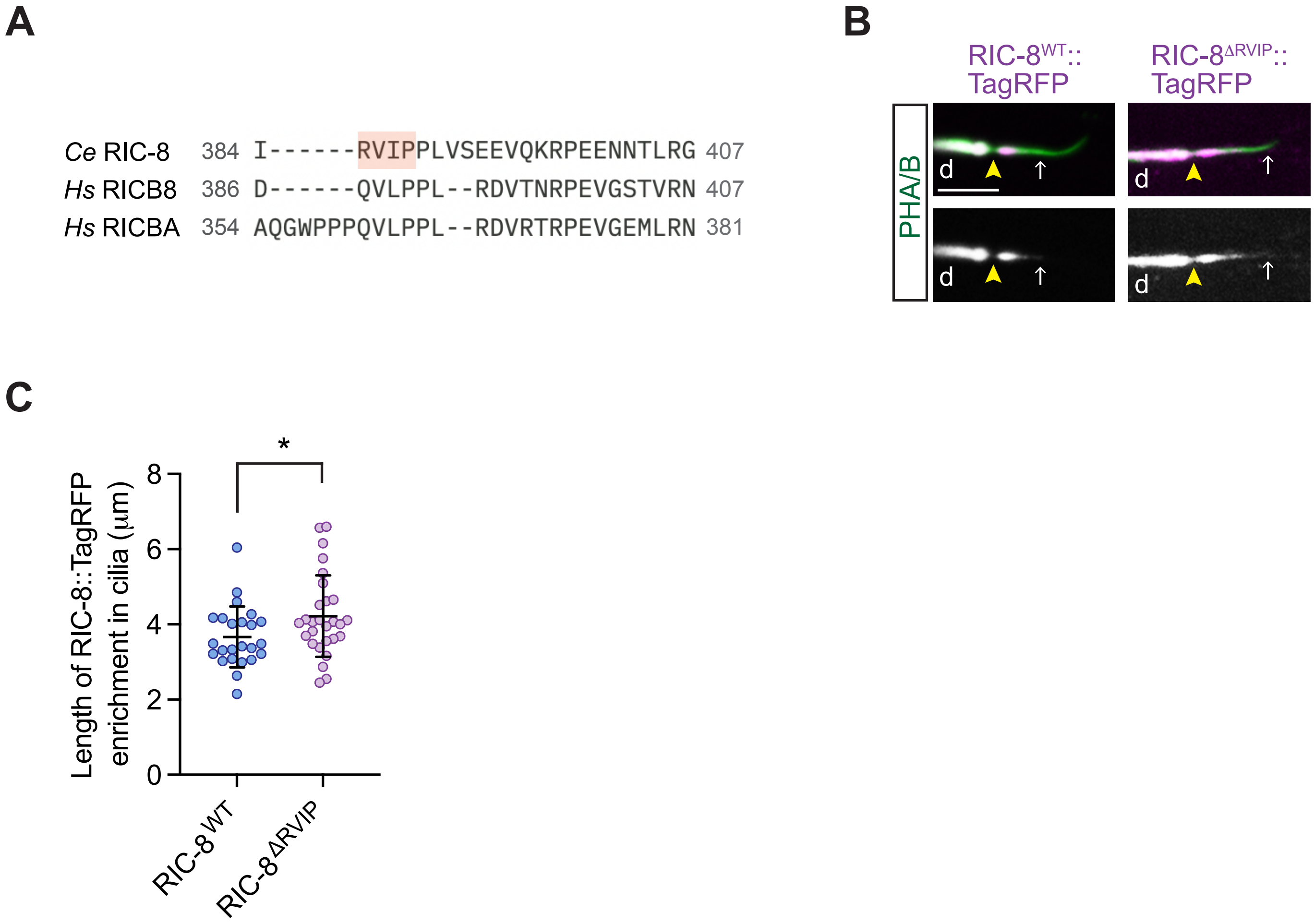
A
Ce RIC-8 384 I------RVIPPLVSEEVQKRPEENNTLRG 407
Hs RICB8 386 D------QVLPPL--RDVTNRPEVGSTVRN 407
Hs RICBA 354 AQGWPPPQVLPPL--RDVRTRPEVGEMLRN 381
B
RIC-8WT::TagRFP
RIC-8ΔRVIP::TagRFP
PHA/B
d
C
Length of RIC-8::TagRFP enrichment in cilia (µm)
*
0
2
4
6
8
RIC-8 WT
RIC-8ΔRVIP

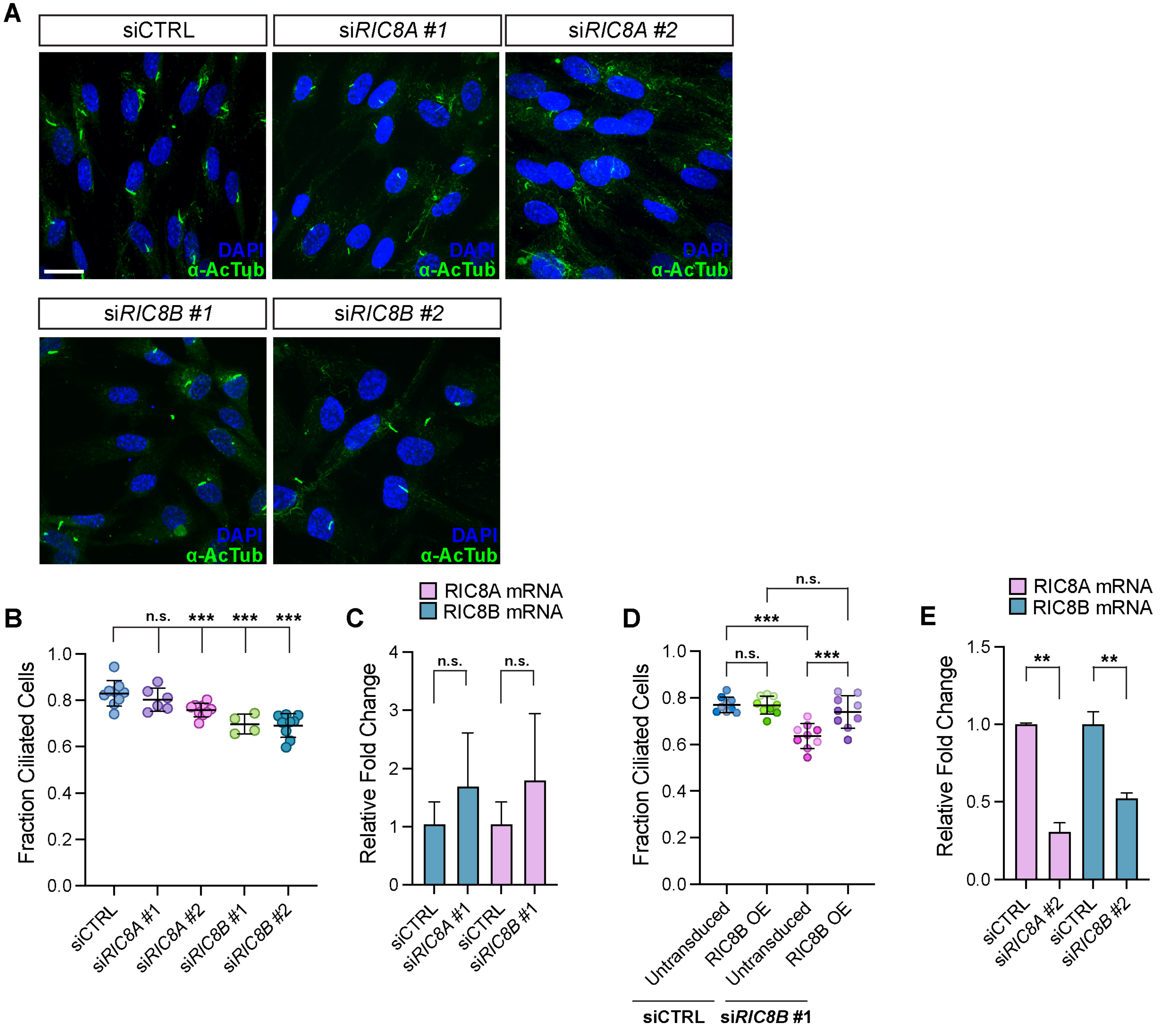
A
siCTRL
siRIC8A #1
siRIC8A #2
DAPI
α-AcTub
siRIC8B #1
siRIC8B #2
B
n.s.
***
Fraction Ciliated Cells
1.0
0.8
0.6
0.4
0.2
0.0
siCTRL
siRIC8A #1
siRIC8A #2
siRIC8B #1
siRIC8B #2
C
RIC8A mRNA
RIC8B mRNA
Relative Fold Change
4
3
2
1
0
D
Untransduced
RIC8B OE
siCTRL
siRIC8B #1
E
**
1.5
1.0
0.5
0.0

Supplementary Table 1. List of strains used in this study

Strain	Genotype	Source
NWM740	ric-8(nch016[ric-8::wrmScarlet$_{11}$]) IV; nchEx018[bbs-8p::wrmscarlet$_{1\text{-}10}$, unc-122Δp::gfp]	This work
NWM029	nchEx004[bbs-8p::ric-8::tagrfp, unc-122p::gfp]	This work
NWM067	nphp-2(gk653) V; nchEx004[bbs-8p::ric-8::tagrfp, unc-122Δp::gfp]	This work
NWM391	mks-5(tm3100) II; nchEx002[bbs-8p::myrgfp, bbs-8p::ric-8::tagrfp, unc-122Δp::gfp]	This work
NWM331	kap-1(ok676) II; nchEx002[bbs-8p::myrgfp, bbs-8p::ric-8::tagrfp, unc-122Δπ::gfp]	This work
NWM168	klp-11(tm324) IV; nchEx002[bbs-8p::myrgfp, bbs-8p::ric-8::tagrfp, unc-122Δp::gfp]	This work
NWM291	xbx-1(ok279) V; nchEx002[bbs-8p::myrgfp, bbs-8p::ric-8::tagrfp, unc-122Δp::gfp]	This work
NWM310	che-3(p801) I; nchEx002[bbs-8p::myrgfp, bbs-8p::ric-8::tagrfp, unc-122Δp::gfp]	This work
NWM644	dyf-2(m160) III; nchEx002[bbs-8p::myrgfp, bbs-8p::ric-8::tagrfp, unc-122Δp::gfp]	This work
NWM355	che-11(e1810) V; nchEx002[bbs-8p::myrgfp, bbs-8p::ric-8::tagrfp, unc-122Δp::gfp]	This work
NWM639	daf-10(p821) IV; nchEx002[bbs-8p::myrgfp, bbs-8p::ric-8::tagrfp, unc-122Δp::gfp]	This work
NWM616	ifta-1(nx61) X; nchEx002[bbs-8p::myrgfp, bbs-8p::ric-8::tagrfp, unc-122Δp::gfp]	This work
NWM365	dyf-1(mn335) I; nchEx002[bbs-8p::myrgfp, bbs-8p::ric-8::tagrfp, unc-122Δp::gfp]	This work
NWM370	dyf-13(mn396) II; nchEx002[bbs-8p::myrgfp, bbs-8p::ric-8::tagrfp, unc-122Δp::gfp]	This work
NWM628	dyf-6(m175) X; nchEx002[bbs-8p::myrgfp, bbs-8p::ric-8::tagrfp, unc-122Δp::gfp]	This work
NWM319	ift-74(tm2394) II; nchEx002[bbs-8p::myrgfp, bbs-8p::ric-8::tagrfp, unc-122Δp::gfp]	This work
NWM315	ift-20(ok3191) I; nchEx002[bbs-8p::myrgfp, bbs-8p::ric-8::tagrfp, unc-122Δp::gfp]	This work
NWM627	che-2(e1033) X; nchEx002[bbs-8p::myrgfp, bbs-8p::ric-8::tagrfp, unc-122Δp::gfp]	This work
NWM625	dyf-11(mn392) X; nchEx002[bbs-8p::myrgfp, bbs-8p::ric-8::tagrfp, unc-122Δp::gfp]	This work
NWM299	osm-3(p802) IV; nchEx002[bbs-8p::myrgfp, bbs-8p::ric-8::tagrfp, unc-122Δp::gfp]	This work
NWM726	dyf-5(ok1177) I; nchEx002[bbs-8p::myrgfp, bbs-8p::ric-8::tagrfp, unc-122Δp::gfp]	This work
NWM643	odr-3(nch013[odr-3::wrmscarlet11]) V; nchEx017[sra-6p::wrmscarlet1-10, unc-122Δp::gfp]	This work
NWM677	ric-8(md1909) IV; odr-3(nch013[odr-3::wrmscarlet11]) V; nchEx017[sra-6p::wrmscarlet1-10, unc-122Δp::gfp]	This work

MOS88	*etyIs1[gpa-13*p*::FLPase, sra-6*p*::FRT::mCherry::Stop::FRT::GCaMP6s]; him-5(e1490) V*	(Pechuk et al., 2022)
MOY127	*etyIs1[gpa-13*p*::FLPase, sra-6*p*::FRT::mCherry::Stop::FRT::GCaMP6s]; ric-8(md1909) IV*	This work

References

Pechuk, V., G. Goldman, Y. Salzberg, A.H. Chaubey, R.A. Bola, J.R. Hoffman, M.L. Endreson, R.M. Miller, N.J. Reger, D.S. Portman, D.M. Ferkey, E. Schneidman, and M. Oren-Suissa. 2022. Reprogramming the topology of the nociceptive circuit in C. elegans reshapes sexual behavior. *Curr Biol*. 32:4372-4385 e4377.
