# Supplementary material for: Functions and trafficking mechanisms of RIC-8 in C. elegans and mammalian cilia
Source: Mol Biol Cell. 2026 Jul 27;37(8):br26. doi: 10.1091/mbc.E25-12-0598 (PMC13428341; doi:10.1091/mbc.E25-12-0598)
Supplement: Supplementary file 2 [file reviewer_reportE25-12-0598.pdf]

Functions and trafficking mechanisms of RIC-8 in C. elegans and mammalian cilia

Inna Nechipurenko, Christina Campagna, Abigail Descoteaux, Abigail Poole, Eric Peet, Nawaphat Malaiwong, and Michael O'Donnell

Corresponding author(s): Inna Nechipurenko, Worcester Polytechnic Institute

Review Timeline:

Submission Date:	2025-12-18
Editorial Decision:	2026-01-21
Revision Received:	2026-06-03
Accepted:	2026-06-08

Editor-in-Chief: John Aitchison

Transaction Report:

(Note: With the exception of the correction of typographical or spelling errors that could be a source of ambiguity, letters and reports are not edited. The original formatting of letters and referee reports may not be reflected in this compilation.)

RE: Manuscript #E25-12-0598
TITLE: Cell-specific roles for the conserved Galpha chaperone RIC-8 in cilia biology

Dear Dr. Nechipurenko:

Thank you for sending this manuscript to MBoC. This is an important body of work and provides critical new insight into the tissue-specificity of RIC-8 function in cilia. We and the reviewers appreciate the care and precision of this study. As you can see in the comments below, the reviewers were positive about the work, having only relatively minor concerns. These likely can be addressed by mostly textual edits and figure revisions to improve the clarity, and perhaps one control experiment. We would be happy to re-evaluate the revised version.

Sincerely,
Rachel E. Turn
Early Career Editor
Gregory Pazour
Monitoring Editor
Molecular Biology of the Cell

Dear Dr. Nechipurenko,

The review of your manuscript, referenced above, is now complete. The Monitoring Editor has decided that your manuscript is not acceptable for publication at this time, but may be deemed acceptable after specific revisions are made, as described in the Monitoring Editor's decision letter above and the reviewer comments below.

A reminder: Please do not contact the Monitoring Editor directly regarding your manuscript. If you have any questions regarding the review process or the decision, please contact the MBoC Editorial Office (mboc@ascb.org).

When submitting your revision include a rebuttal letter that details, point-by-point, how the Monitoring Editor's and reviewers' comments have been addressed. (The file type for this letter must be "rebuttal letter"; do not include your response to the Monitoring Editor and reviewers in a "cover letter.") Please bear in mind that your rebuttal letter will be published with your paper if it is accepted, unless you have opted out of publishing the review history.

Authors are allowed 90 days to submit a revision. If this time period is inadequate, please contact us at mboc@ascb.org.

Revised manuscripts are assigned to the original Monitoring Editor whenever possible. However, special circumstances may preclude this. Also, revised manuscripts are often sent out for re-review, usually to the original reviewers when possible. The Monitoring Editor may solicit additional reviews if it is deemed necessary to render a completely informed decision.

In preparing your revised manuscript, please follow the instruction in the Information for Authors (www.molbiolcell.org/info-for-authors). In particular, to prepare for the possible acceptance of your revised manuscript, submit final, publication-quality figures with your revision as described.

To submit the rebuttal letter, revised manuscript, and figures, use this link: Link Not Available

MBoC offers the option to publish your work with immediate open access. Open access can increase the discoverability and usability of your research. If you would like to publish your paper with immediate open access but did not select this option during initial submission, please contact the editorial office at mbc@ascb.org.

Authors of Articles and Brief Communications are also encouraged to create a short video abstract to accompany their article when it is published. These video abstracts, known as Science Sketches, are up to 2 minutes long and will be published on YouTube and then embedded in the article abstract. Science Sketch Editors on the MBoC Editorial Board will guide you as you prepare your video. Information about how to prepare and submit a video abstract is available at www.molbiolcell.org/science-sketches. Please contact mboc@ascb.org if you are interested in creating a Science Sketch.

Sign up for MBoC's new content alerts here https://tinyurl.com/3bcsyk3e

Thank you for submitting your manuscript to MBoC. We look forward to receiving your revised paper.

Sincerely,

Eric Baker
Journal Production Manager
MBoC Editorial Office
mbc@ascb.org

Reviewer #1 (Remarks to the Author):

In Campagna et al's article, "Cell-specific roles for the conserved Galpha chaperone 1 RIC-8 in cilia biology," the authors provide critical new insight into the tissue-specific functions of the Galpha GEF RIC8 in driving ciliogenesis and key mechanisms that are required for its proper localization and function. Furthermore, this work is instrumental for understanding the mechanisms governing the function of the inversin compartment and the interplay of different mechanisms governing the compartment specificity. Below, we summarize the key takeaways from the manuscript:

Summary of major points from article:
• RIC-8 becomes restricted to the InvC during larval development independently of NPHP-2. The authors used a split-Scarlett reporter system in C. elegans to assess RIC-8 localization to cilia in different stages of development. These data reveal context-specificity of RIC-8 localization, namely that it becomes sequestered to the inversin compartment later in development. These data are pivotal, providing insight into different protein functionality across the developmental landscape.

• The transition zone regulates intraciliary distribution of RIC-8 in channel cilia
Here, the authors demonstrate the importance of maintaining a functional transition zone in preventing loss of RIC-8 from the inversin compartment. These data further highlight the importance of the transition zone in maintaining the integrity of the primary cilium and preventing the spurious "leaking" of key components, including RIC-8.

• The RVxP motif helps restrict RIC-8 to the InvC
The authors test the function of RIC-8's RVxP motif by mutating it and testing localization. Surprisingly, RIC-8 still localizes to primary cilia, but distributes throughout the primary cilium. These data are important, as it reflects the context-specificity of the RVxP motif and that other factors may be at play for ensuring RIC-8 ciliary localization.

• RIC-8 ciliary localization is dependent on IFT
Together, these data reveal different roles for different IFT components in targeting RIC-8 to the primary cilium. The authors do detailed work of tracking the roles of different motors and IFT components in RIC-8 localization and function, working carefully to dissect differences in abundance due to ciliary length versus actual effects on localization and ciliary abundance.

• DYF-5 restricts RIC-8 to the InvC
Furthermore, the authors demonstrate how the DYF-5 kinase helps regulate RIC-8 localization to InvC. It may be interesting in the future (and an area that the authors might comment on) to identify which substrates DYF-5 is regulating to alter RIC-8 functionality.

• ric-8 mutants are defective in glycerol responses mediated by ASH neurons
The authors tie in nicely how defects in ric-8 function led to altered sensory response of ASH neurons in C. elegans. Showing similar level of defects in response to glycerol exposure as mutations in Galpha. It would be interesting to which receptors are defective upon RIC-8 disruption and which cell types.

• Human RIC8A and RIC8B contribute to ciliogenesis in RPE-1 cells.
These data reveal critical roles of both RIC8A and RIC8B in regulating ciliogenesis in RPE-1, with RIC8B having a larger defect. As noted below, it would be interesting to decipher how much RIC8A and RIC8B have overlapping functions

Altogether, this is an excellent work that provides molecular details into the tissue specificity and the context specificity of RIC-8 functionality. Below, we provide a few suggestions on ways to expand the impact of the already great work performed by this team.
Major Comments:
1. It would be very helpful to the reader to know the greater biological implications of RIC-8 to human health. Does its mutation/disruption lead to classic ciliopathy phenotypes?
2. For the RIC-8B shown in Figure 5A, it looks like the intensity of ARL13B at cilia is diminished, as well as it looked like ciliary length is decreased. Is this the case?
3. The use of rescue controls would be useful to support the rigor of these studies. It would also be interesting to see the degree with which over-expression of RIC-8A versus RIC-8B can rescue the loss of the other, therefore reflecting the degree to which they have overlapping functions.
Minor Comments:

1. Is it known which Galphas that RIC8 regulates? Also, what is the difference in Galpha expression and GPCRs that are functioning in channel versus wing cilia in worms? A minor point, but it would be interesting to know considering the cell type specificity found here.

Reviewer #2 (Remarks to the Author):

In their manuscript, "Cell-specific roles for the conserved Galpha chaperone RIC-8 in cilia biology," Campagna et al use in vivo and in vitro approaches to investigate RIC8 distribution in cilia and the consequence of RIC8 mutants on neuronal signaling. Using C. elegans sensory neurons, the authors explain the developmental and molecular mechanisms that govern RIC-8 targeting to the inversin compartment of channel cilia, identifying contributions from the transition zone, an RVxP targeting motif, and intraflagellar transport proteins. In this elegant genetic study, the authors use several crosses to thoroughly examine RIC-8 localization and its dependency on motor proteins/machinery in vivo. The study further demonstrates a functional requirement for RIC-8 in ASH-mediated chemosensory signaling through the regulation of Gα protein levels and neuronal responses. In addition, the authors extend these findings to mammalian cells, showing that human RIC8A and RIC8B are required for efficient ciliogenesis in RPE-1 cells. Overall, this work provides new insight into ciliary subdomain organization and highlights conserved and context-dependent functions of RIC-8 in sensory cilia. Some comments to improve clarity and ensure the conclusions have sufficient support from the data include:
1. The title could be clarified to better reflect the scope and focus of the study. The manuscript is framed as being cell-specific, but only a small number of cell types are examined.
2. The manuscript focuses on the chaperone role of RIC-8, but the GEF activity of RIC-8 is largely ignored. The authors should justify this in the manuscript. Do we know which GTPase(s) RIC-8 activates? If so, do mutants of those GTPases phenocopy the loss of RIC-8 phenotype? Addressing this would help with the interpretation of the results. [Updating this thought/comment after further research; Campagna et al 2023 shows the rescue of RIC-8 mutants depends on the chaperone activity and not GEF activity which justifies the focus on the chaperone role in this study. This should be made clearer to the reader to highlight why one role was the focus over the other].
3. The main emphasis of this study is on where RIC-8 localizes under different conditions rather than what RIC-8 is doing. The localization data is extensive, but the change in function or mechanisms as a result is less clear and would benefit from further discussion.
4. Some clarifications regarding the data presented in Figure 1:
The staining in Fig 1B and 1C do not match. RIC-8 appears to localize to the InvC in 1C but not in 1B, and this is not explained/discussed. The description of the length of RIC-8 enrichment is not fully accurate. There is clear enrichment of RIC-8 at the distal tip of L1 larvae, with most signal in the PCMC. Also, some images show RIC-8 extending distal to the transition zone, while others show it absent from that region.
5. Images of young adults are not shown in Fig 1C (to accompany Fig 1D/E), making it difficult to compare young adults to L1 larvae. It is also unclear how Fig 1G/H relate to 1D/E, as they appear to show different things. Are they presented out of order? Showing changes in RIC-8 distribution using a plot similar to Figure 2C may better represent these differences.
6. In Fig 2c, mks-5 mutants appear to have more RIC-8 in cilia due to how the data is presented, which is contradictory to the actual results. Normalizing RIC-8 intensity to the PCMC region or the distal dendrite rather than to total signal may help clarify this result.
7. In Fig 3C, white arrows in both the top and bottom panels may improve clarity.
8. In Fig 3D, individual data points and error bars should be shown to better represent the data.
9. Prior work (Campagna et al., 2023) analyzed two different RIC-8 mutant alleles, but the manuscript only investigates RIC-8 md1909. Although this allele is chaperone-deficient, this is not clearly stated in the manuscript and should be explained.

Reviewer #3 (Remarks to the Author):

The study by Campagna et al. examines molecular determinants of RIC-8 intraciliary distribution, specifically its restriction to the InvC compartment, named after the NPHP2 (Inversin) gene. The proper function of many ciliary proteins critically depends on their strict localization to a particular ciliary region, and when disrupted, often leads to the malfunction of the whole cilium. The study is a step forward toward our understanding of the molecular logic of sub-ciliary compartment organization. Authors show that RIC-8 restriction to the proximal region is independent of nphp-2 and that its levels are regulated by ciliary motors (kinesin-3 and dynein-2), the IFT machinery, and MAK/ICK kinase DYF-5. Deletion of the inversin-targeting sequence (RVIP) in the RIC-8 protein resulted in the protein leakage to the distal regions of the cilium. Authors identified that RIC-8 functions in the ASH cilium, regulating the levels of ODR-3 (a G-protein alpha subunit) required for the response to a hyperosmotic challenge, such as 1M glycerol. Consistent with this, the ASH neurons of ric-8 mutants had reduced Ca^{2+} spiking in response to 1M glycerol exposure. Authors also show that knockdown of RIC8A and RIC8B reduced the percentage of ciliated cells in the RPE cell culture, although the effect appears mild, perhaps due to residual RIC8 mRNA levels or reduced RIC8A and RIC8B protein turnover.
The study's greatest strength is the use of endogenous reporters in an in vivo model, which allows assessment of RIC-8

compartmentalization and function under the most native conditions possible. Overall, the manuscript is clear, and all findings support the claims.

Minor comments:
Please indicate in all figure legends which cilia are shown/quantified on which panel.

Response to Reviewers

We would like to thank the reviewers for their thoughtful and insightful comments that helped us improve the quality and clarity of the manuscript. Our responses to reviewers' comments are summarized below.

<u>**Reviewer 1**</u>
<u>Major points:</u>

> *1. It would be very helpful to the reader to know the greater biological implications of RIC-8 to human health. Does its mutation/disruption lead to classic ciliopathy phenotypes?*

To our knowledge, neither *RIC8A* nor *RIC8B* has been directly linked to ciliopathies; although >30 variants of uncertain significance have been deposited in the ClinVar database for each gene. Interestingly, global knockout of *ric8a* or *ric8b* in the mouse model results in early embryonic lethality (PMID:22114146, 21069829), similarly to knockouts of the core cilia genes.

> *2. For the RIC-8B shown in Figure 5A, it looks like the intensity of ARL13B at cilia is diminished, as well as it looked like ciliary length is decreased. Is this the case?*

We have replaced the image of cells treated with si*RIC8B* in Figure 5A with a more representative image.

We did quantify cilia length in RPE-1 cells treated with si*RIC8B* siRNAs (see below). As there was no significant difference in cilia length between siControl and si*RIC8B*-treated cells, we did not include these data in the manuscript due to space limitations.

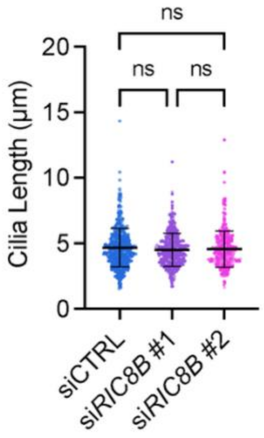

Fig. 1. Cilia length is normal in *RIC8B* KD cells. Quantification of ciliation in RPE-1 cells transfected with the indicated siRNAs. The data represent three biological replicates with three technical replicates each. ns: not significant (Kruskal-Wallis test).

> *3. The use of rescue controls would be useful to support the rigor of these studies. It would also be interesting to see the degree with which over-expression of RIC-8A versus RIC-8B can rescue the loss of the other, therefore reflecting the degree to which they have overlapping functions.*

Thank you for this suggestion to improve the rigor of our manuscript. *We have carried out a rescue experiment by overexpressing RIC8B cDNA refractory to RNAi using lentivirus in the RIC8B KD cells* (see new Supplemental figure 2D). We chose to rescue the *RIC8B*- rather than *RIC8A*-dependent ciliation phenotype because decrease in ciliation following *RIC8B* KD was more dramatic compared to *RIC8A* KD (Figure 5, A and B; Supplemental figure 2, A and B), which could be due to off-target effects of RNAi.

Regarding the reviewer's second point: mammalian RIC-8 homologs (RIC8A and RIC8B) are generally believed to work with distinct Gα protein clients based on extensive *in vivo* and structural data. Specifically, RIC8A binds and regulates Gαi/o, Gαq, and Gα12/13 proteins, while RIC8B has exclusive preference for Gαs/olf (e.g. PMID:12509430, 20133939, 15829631, 21467038), arguing against overlapping functions. *We now note this in the body of the manuscript*. Consistently, in our cell culture experiments, partial KD of either *RIC8A* or *RIC8B* alone causes ciliation defects (albeit to a different extent) (Fig 5 and Supplemental figure 2), suggesting that RIC8A cannot fully compensate for reduced levels of RIC8B and *vice versa* in this cellular context.

Finally, to ensure that si*RIC8A* does not also non-specifically KD *RIC8B* and *vice versa*, we quantified *RIC8B* transcript levels following *RIC8A* KD and *vice versa* (see new Supplemental figure 2C).

<u>Minor point:</u>

> *1. Is it known which Galphas that RIC8 regulates? Also, what is the difference in Galpha expression and GPCRs that are functioning in channel versus wing cilia in worms? A minor point, but it would be interesting to know considering the cell type specificity found here.*

Please see response to point #3 above regarding Gα protein specificity of the mammalian RIC8A and RIC8B. Since there is only one *ric-8* gene in *C. elegans*, it is possible that *C. elegans* RIC-8 regulates all classes of Gα proteins. Alternatively, worm RIC-8 may bind and regulate only a subset of Gα proteins. Future work is needed to systematically test RIC-8 binding to and regulation of *C. elegans* Gα proteins.

<u>Reviewer 2:</u>

1. *The title could be clarified to better reflect the scope and focus of the study. The manuscript is framed as being cell-specific, but only a small number of cell types are examined.*

Done.

2. *The manuscript focuses on the chaperone role of RIC-8, but the GEF activity of RIC-8 is largely ignored. The authors should justify this in the manuscript. Do we know which GTPase(s) RIC-8 activates? If so, do mutants of those GTPases phenocopy the loss of RIC-8 phenotype? Addressing this would help with the interpretation of the results. [Updating this thought/comment after further research; Campagna et al 2023 shows the rescue of RIC-8 mutants depends on the chaperone activity and not GEF activity which justifies the focus on the chaperone role in this study. This should be made clearer to the reader to highlight why one role was the focus over the other].*

Please see response to reviewer 1, point #3 regarding G protein clients of RIC-8. We also showed in (PMID: 37910589) that *C. elegans* RIC-8 regulates Gα ODR-3, and that *ric-8* lof

mutants phenocopy *odr-3* lof mutants with respect to cilia morphology in AWC and ASH (see also PMID: 41052774) neurons as well as sensory signaling deficits (current manuscript and references therein). *We have clarified this background information in the manuscript*. Additionally, since RIC8 homologs are required for Gα protein folding and their targeting to membranes (as Gα chaperones), it has been notoriously challenging to uncouple the GEF and chaperone functions of RIC8 proteins *in vivo*.

3. *The main emphasis of this study is on where RIC-8 localizes under different conditions rather than what RIC-8 is doing. The localization data is extensive, but the change in function or mechanisms as a result is less clear and would benefit from further discussion*.

We agree with the reviewer's point that it will be important to investigate whether localization to the InvC is essential for RIC-8 function. Since RIC-8 localization to the cilium is regulated by IFT and the transition zone, which mediate trafficking of many other ciliary proteins besides RIC-8, addressing this question would require: 1) identifying RIC-8 residues that are necessary for its trafficking to the cilium via IFT and/or TZ-dependent mechanisms and 2) generating corresponding *ric-8* alleles. Similarly, *ric-8* mutants with the deleted RVIP motif, which is important for restricting RIC-8 to the InvC, could be generated to determine if changes in RIC-$8^{\Delta RVIP}$ localization are accompanied by sensory deficits. These experiments are beyond the scope of this brief report and constitute an exciting future direction. *We now added a statement acknowledging these experiments as an important future direction to the relevant section of the manuscript*.

4. *Some clarifications regarding the data presented in Figure 1:*
   The staining in Fig 1B and 1C do not match. RIC-8 appears to localize to the InvC in 1C but not in 1B, and this is not explained/discussed. The description of the length of RIC-8 enrichment is not fully accurate. There is clear enrichment of RIC-8 at the distal tip of L1 larvae, with most signal in the PCMC. Also, some images show RIC-8 extending distal to the transition zone, while others show it absent from that region.

We replaced images in Fig 1B and 1C with more representative images and added arrows to more clearly mark distal boundaries of RIC-8 signal inside the cilia in Fig 1 panels A-C. In L1 larvae, RIC-8 appears to be enriched in the proximal cilium (distal to the TZ), but it also extends beyond the InvC into more distal cilia segments, as evident from the new Figure 1C, which shows RIC-8 colocalization with the InvC marker NPHP-2 in L1 larvae and adults.

5. *Images of young adults are not shown in Fig 1C (to accompany Fig 1D/E), making it difficult to compare young adults to L1 larvae. It is also unclear how Fig 1G/H relate to 1D/E, as they appear to show different things. Are they presented out of order? Showing changes in RIC-8 distribution using a plot similar to Figure 2C may better represent these differences.*

We added an image of an adult animal to Fig. 1C.

Panels 1D and E present quantification of RIC-8::TagRFP distribution inside the cilia of wild type L1 larvae and young adults using two different methods: as length of RIC-8 signal inside the cilium, with each data point representing a length measurement from one cilium (Fig 1D), and as a ratio of RIC-8 signal length to the length of the cilium, with each data point representing a ratio from a single cilium (Fig 1E) to illustrate the point that RIC-8 occupies a larger fraction of the cilium in L1 larvae compared to adults.

We swapped panels 1G and 1H to improve clarity of the narrative. The purpose of panel 1G is to show that RIC-8 levels inside the cilia of *nphp-2* mutants (relative to distal dendrite) are not altered, therefore, suggesting that RIC-8 localization to the cilium is not dependent on NPHP-2. The purpose of panel 1H is to show that RIC-8 distribution inside the cilium (i.e. length of RIC-8 signal) is also not affected in *nphp-2* mutants (i.e. RIC-8 is still restricted to the proximal ciliary segment). *We edited the text and figure legends for improved clarity*.

6. *In Fig 2c, mks-5 mutants appear to have more RIC-8 in cilia due to how the data is presented, which is contradictory to the actual results. Normalizing RIC-8 intensity to the PCMC region or the distal dendrite rather than to total signal may help clarify this result.*

Quantification in Fig. 2b shows exactly that – RIC-8::TagRFP intensity inside the cilium normalized to the PCMC region.

Fig. 2c is meant to illustrate differences in RIC-8::TagRFP intra-ciliary distribution between wild type and *mks-5* mutants. To this end, TagRFP intensity was measured inside each cilium along a straight line (in Fiji/Image J) starting from the transition zone. Then, the intensity at each point along this line was normalized to the maximum intensity value within the same cilium. *We clarified description of the quantification method in the Image analysis section of Methods.* The resultant data from Fig. 2C indicate that RIC-8::TagRFP signal is more evenly distributed throughout the measured ciliary region of *mks-5* mutants compared to wild type, where RIC-8::TagRFP signal peaks around 1μm distal to the transition zone and drops more distally.

7. *In Fig 3C, white arrows in both the top and bottom panels may improve clarity.*

Done.

8. *In Fig 3D, individual data points and error bars should be shown to better represent the data.*

Fig. 3D shows the percentage of ASH cilia that have RIC-8::TagRFP extending to the cilia tip. The total number of animals examined is noted inside the bars.

9. *Prior work (Campagna et al., 2023) analyzed two different RIC-8 mutant alleles, but the manuscript only investigates RIC-8 md1909. Although this allele is chaperone-deficient, this is not clearly stated in the manuscript and should be explained.*

In Campagna *et al*., 2023, we analyzed two hypomorphic alleles (*md303* and *md1909*) and one null allele (*ok98*) of *ric-8* and found that all three alleles exhibited qualitatively and quantitatively

similar defects in cilia morphology, similarly to their effects on synaptic function published by others (PMID: 10985349). We chose to use hypomorphic alleles for subsequent analyses in Campagna *et al*., 2023 and in the current manuscript out of convenience, because *ric-8* null animals do not complete development and arrest as L3 larvae, complicating morphological and functional analyses of cilia. *We now specifically state in the manuscript that we are using a hypomorphic allele of ric-8.*

Reviewer 3

Minor point:

Please indicate in all figure legends which cilia are shown/quantified on which panel.

Done.

RE: Manuscript #E25-12-0598R
TITLE: "Functions and trafficking mechanisms of RIC-8 in C. elegans and mammalian cilia"

Dear Dr. Nechipurenko:

I am pleased to accept your manuscript for publication in Molecular Biology of the Cell.

Thanks for sending this interesting work to MBoC! Both Dr Turn and I appreciate the effort that you made to address the concerns of the reviewers and feel that this paper is a strong contribution to the field.

Sincerely,
Rachel E. Turn
Early Career Editor
Gregory Pazour
Monitoring Editor
Molecular Biology of the Cell

Dear Dr. Nechipurenko:

Congratulations on the acceptance of your manuscript! Thank you for publishing your work in Molecular Biology of the Cell (MBoC).

Within 10 days, an unedited PDF of your manuscript will be published on MBoC in Press, an early-release journal version. The date your manuscript appears on this site is the official publication date.

Your copyedited and typeset manuscript will be scheduled for publication in the next available issue of MBoC. Once your paper is ready for review, our production team will notify you. In the summer of 2024, our production provider will introduce an online proofing solution, which will streamline the process of checking and validating author changes. This will result in improved typesetting quality, enhanced digital content, and a faster overall process.

Your paper is among those chosen by the Editorial Board for Highlights from MBoC. Hightlights from MBoC appears in the ASCB Newsletter and highlights the important articles from the most recent issue of MBoC.

All Highlights papers are also considered for the MBoC Paper of the Year. In order to be eligible for this award, however, the first author of the paper must be a student or postdoc. Please email me to indicate if this paper is eligible for Paper of the Year.

We invite you to submit images related to your accepted manuscript to be considered for the journal cover.
Cover images must contain color, TIF Format
Size image at 7.4" wide x 5.85" deep
RGB and CMYK color (please submit two separate files, one in each format)
300 dpi resolution

Please also send a 1-paragraph description of the image that a general audience could understand. Include with the caption the name and institution of the individual(s) the image should be attributed to. Please provide this description in a Word file. You can send the file via email or by your favorite file transfer service to mboc@ascb.org.

Authors of Articles and Brief Communications are also encouraged to create a short video abstract to accompany their article when it is published. These video abstracts, known as Science Sketches, are up to 2 minutes long and will be published on YouTube and then embedded in the article abstract. Science Sketch Editors on the MBoC Editorial Board will guide you as you prepare your video. Information about how to prepare and submit a video abstract is available at www.molbiolcell.org/science-sketches. Please contact mboc@ascb.org if you are interested in creating a Science Sketch.

We look forward to publishing your paper in MBoC.

Sincerely,

Eric Baker
Journal Production Manager
MBoC Editorial Office
mbc@ascb.org

Reviewer #1 (Remarks to the Author):

In Campagna et al's article, "Functions and trafficking mechanisms of RIC-8 in C. elegans and mammalian cilia," the authors provide critical new insight into the mechanisms governing RIC-8 function at the inversin compartment, highlighting the importance of sub-ciliary compartment organization for overall ciliary function in both C. elegans and mammalian tissue culture. The authors do an excellent job at addressing reviewer comments, completing the control experiment requested, improving figure labeling, and providing more detailed explanations in the text.
